# Effects of Oral Anthocyanin Supplementation on In Vitro Neurogenesis, Hippocampus-Dependent Cognition, and Blood-Based Dementia Biomarkers: Results from a 24-Week Randomized Controlled Trial in Older Adults At Risk for Dementia (ACID)

**DOI:** 10.3390/nu17162680

**Published:** 2025-08-19

**Authors:** Chiara de Lucia, Diego Alejandro Tovar-Rios, Khadija Khalifa, Silje Meihack Kvernberg, Ilaria Pola, Anne Katrine Bergland, Jodi Maple-Grødem, Richard Siow, Nicholas Ashton, Clive Ballard, Sandrine Thuret, Dag Aarsland

**Affiliations:** 1 Centre for Age-Related Medicine (SESAM), Stavanger University Hospital, Armauer Hansens vei 30, 4011 Stavanger, Norway; 2 Department of Psychological Medicine, Institute of Psychiatry, Psychology and Neuroscience, King’s College London, Memory Lane, London S5 9NU, UK; 3 Department of Basic and Clinical Neuroscience, Maurice Wohl Clinical Neuroscience Institute, Institute of Psychiatry, Psychology and Neuroscience, King’s College London, 125 Coldharbour Lane, London SE5 9NU, UK; 4 Doctoral School Biomedical Sciences, Katholieke Universiteit, Herestraat 49, P.O. Box 700, 3000 Leuven, Belgium; 5 Grupo de Investigación en Estadística Aplicada—INFERIR, Universidad del Valle, Cali 760032, Colombia; 6 Prevención y Control de la Enfermedad Crónica—PRECEC, Universidad del Valle, Cali 760032, Colombia; 7 The Faculty of Health Sciences, University of Stavanger, Telegrafdirektør Heftyes vei 73, 4021 Stavanger, Norway; 8 Department of Psychiatry and Neurochemistry, Institute of Neuroscience and Physiology, The Sahlgrenska Academy, University of Gothenburg, Wallinsgatan 6, 431 39 Mölndal, Sweden; 9 Department of Clinical Medicine, University of Bergen, Haukelandsveien 28, 5009 Bergen, Norway; 10 The Centre for Movement Disorders, Stavanger University Hospital, Armauer Hansens vei 30, 4011 Stavanger, Norway; 11 Department of Chemistry, Bioscience and Environmental Engineering, University of Stavanger, Kristine Bonnevies vei 22, 4021 Stavanger, Norway; 12 School of Cardiovascular and Metabolic Medicine and Sciences, King’s BHF Centre of Research Excellence, Faculty of Life Sciences & Medicine, King’s College London, 50 Stamford Street, London SE1 9NH, UK; 13 Department of Physiology, Anatomy and Genetics, Medical Sciences Division, University of Oxford, Sherrington Building, Sherrington Road, Oxford OX1 3PT, UK; 14 Banner Alzheimer’s Institute, 901 E Willetta St, Phoenix, AZ 85006, USA; 15 Banner Sun Health Research Institute, 10515 W Santa Fe Drive, Sun City, AZ 85351, USA; 16 Department of Health and Community Sciences, Faculty of Life Sciences, University of Exeter, Heavitree Road, Exeter EX1 12LU, UK

**Keywords:** neurogenesis, blood-based biomarkers, hippocampal progenitor cells, anthocyanins, cognition

## Abstract

Background: Identifying compounds with neuroprotective properties that target the neurogenic process will have a considerable impact on dementia prevention. Methods: This is a secondary analysis of a 24-week randomised, double-blind, placebo-controlled anthocyanin supplementation trial in 181 participants. Using blood-derived serum collected during this trial, we treated hippocampal progenitor cells and analysed the ensuing cellular changes in the context of the participant’s clinical and blood-based biomarker data. Results: We show that anthocyanin supplementation impacts hippocampal progenitor cells and that this can impact hippocampal-dependent cognition. We also show for the first time that blood-based dementia biomarkers correlate with human in vitro neurogenesis markers. Conclusions: Our data demonstrates moderator effects of BMI and ApoE4 carrier status and supports the need for more individualised trials. Further studies are warranted to explore the mechanism of action of anthocyanins and the use of blood-based biomarkers for clinical trial enrichment, trial individualization, and therapy development. Trial registration: NCT03419039; date first registered: 15/01/2018.

## 1. Introduction

The development of neurodegenerative diseases like Alzheimer’s disease (AD) is linked to an accumulation of damage caused by oxidative stress and ensuing inflammation [1,2]. Therefore, interventions that target these mechanisms have been suggested as potential prevention strategies. These processes involve different dementias caused by varied risk factors, suggesting that similar preventative strategies could be used across dementias.

Brain tissues and neural stem cells (NSCs) are particularly susceptible to oxidative damage due to high metabolic rates and only modest antioxidant levels. Preventing this damage and the ensuing susceptibility to neurodegeneration is thus a key focus in ageing research.

Of particular interest is adult hippocampal neurogenesis (AHN, i.e., the creation of new neurons from NSC throughout adulthood. AHN has functional effects across hippocampus-dependent cognitive domains, such as those involved in learning and memory, and has been repeatedly implicated in dementia, with markers of neurogenesis being linked to both cognitive diagnosis and Braak staging [3,4]. Neurogenic changes have been detected prior to the deposition of plaques and neurofibrillary tangles, suggesting that they are an integral part of the disease process rather than a consequence [3]. Therefore, identifying compounds that target the neurogenic process could impact disease prevention.

As AHN is heavily modulated by the circulatory system and lifestyle factors [5,6], we established a human in vitro neurogenesis model to test how the circulatory system affects in vitro AHN [7,8]. Using this model, we have shown that varying environmental factors strongly impact AHN [9,10,11,12]. In parallel, antioxidant-rich diets are being explored to delay age-related conditions [13]. Understanding the effects of dietary antioxidants on NSCs and the ensuing AHN will further elucidate the potential of these interventions.

Anthocyanins (ACNs) are some of the better studied dietary antioxidants. ACNs are dietary flavonoids found in dark berries and fruits. They are able to cross the blood–brain barrier and have proven anti-inflammatory properties, making them up-and-coming candidates for dementia prevention [14,15]. Given the role of AHN in dementia and its susceptibility to inflammation and oxidative stress, it has been proposed that ACNs could exert their protective effects via AHN-dependent mechanisms [16,17]. Trials involving the consumption of ACN-rich juices showed protective effects in older individuals with memory impairments, and the intake of ACN-rich food was associated with reduced risk of AD [18,19,20]. The 24-week phase II “AnthoCyanins In people at risk for Dementia” (ACID) randomised placebo-controlled trial was designed to test the effect of ACN supplementation on clinical and biological factors in individuals at risk for dementia [14], and it is the longest such trial supported by blood-based biomarker collection to date.

Here, we present the results of a secondary analysis using biological samples and data collected throughout the ACID trial. We used blood-derived serum collected during this trial to treat hippocampal progenitor cells (HPCs) as part of our in vitro neurogenesis assay to understand the effect of in vivo ACN supplementation on neural stem cell health. We investigate how our in vitro data relate to other relevant biomarkers, such as hippocampus-dependant cognitive performance and blood-based biomarkers (BBMs) of central nervous system health (i.e., GFAP, *p*-tau217, *p*-tau231, and NFL protein levels).

## 2. Materials and Methods

### 2.1. ACID Clinical Trial

This study used serum samples from the double-blind, placebo-controlled randomised clinical trial “ACID” [14,21] (NCT03419039, date first registered: 15 January 2018). Briefly, individuals aged 65 and above at risk for dementia were recruited by 3 Norwegian centres in 2018–2020. Participants were randomised to receive either Medox^®^ capsules, a standardised nutraceutical product containing maltodextrin and 80 mg (320 mg daily) of natural purified ACNs from bilberry (Vaccinium myrtillus) and black currant (Ribes nigrum), or placebo capsules [14]. This secondary study focuses on a subset of participants with available blood-derived serum at baseline and at the endpoint (Table 1 and Table S1).

### 2.2. ApoE Genotyping

Genomic DNA was extracted from peripheral blood using standard methods. Allelic discrimination analysis was performed using predesigned TaqMan SNP genotyping assays for rs7412 and rs429358, as described in [21].

### 2.3. Cognitive Performance

Cognitive performance was assessed using the online cognitive battery CogTrack^®^ [22], which participants completed at both baseline and at the endpoint. For this study, we focused on three measures that are based on picture recognition and thus associated with hippocampal function: Picture Recognition Original Stimuli Accuracy (DPICOACC), Picture Recognition New Stimuli Accuracy (DPICNACC), and a combination “CMB” score of DPICOACC and DPINACC (see Supplementary Materials for further information).

### 2.4. In Vitro Neurogenesis Model

**Figure 1:**
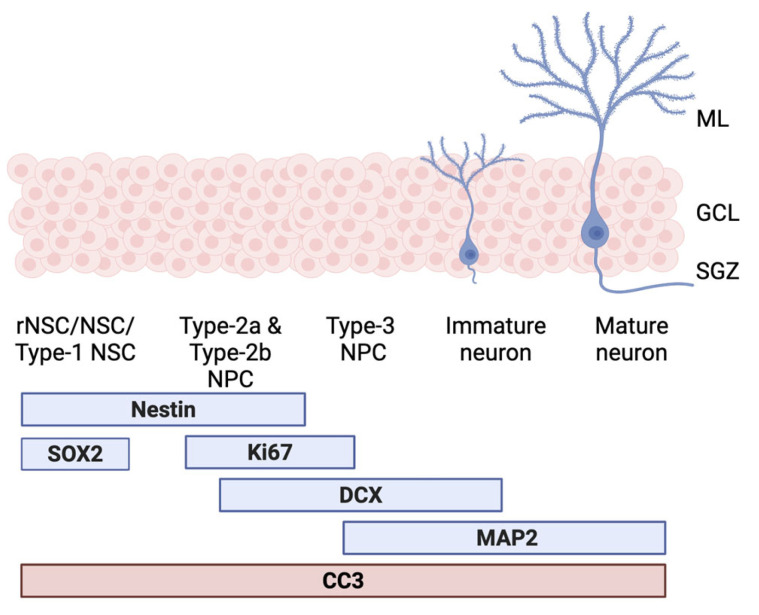
Markers of the neurogenic process. Diagram showing how the selected neurogenesis markers target different aspects of the neurogenic process: starting from neural stem cells (left) through to mature neurons (right). Bars represent the markers expressed by each cell type. These include markers of stem cell integrity (SOX2 and Nestin), a marker of proliferation (Ki67), a marked of neurobalsts (DCX), and a marker of immature neurons (Map2). CC3, a marker of apoptosis can be expressed by any of these cell types. It is an integral part of neurogenesis regulation. rNSCs/NSCs: radial glial-like or neural stem cells; NPCs: neural progenitor cells. ML: molecular layer, GCL: granular cell layer, SGZ: subgranular zone, SOX2: SRY (sex determining region Y)-box 2, Ki67: Antigen Kiel 67, DCX: doublecortin, MAP2: microtubule associated protein 2, and CC3: cleaved caspase 3. Figure adapted from [23] in BioRender.

We used serum collected as part of the ACID trial to treat the human HPC line, HPC03A/07, as part of the neurogenesis assay previously established by the Thuret lab and described elsewhere [7]. The key outcomes of the in vitro assay are ICC markers at various stages of the neurogenic process; plates from the proliferation assay were stained with markers of stem cell integrity (SOX2 and Nestin), with proliferation marker Ki67 (ki67p) and apoptosis marker CC3 (CC3p). Differentiation plates were stained with Ki67 (ki67d) and CC3 (CC3d), as well as with DCX, a marker of neuroblasts, and with MAP2, a marker of immature neurons [10] (see Figure 1).

HPCs were fixed, blocked and stained using primary and secondary antibodies at appropriate dilutions (Table S2). Each plate contained a negative control, media only, and reference serum wells. Table S3 reports the average levels of each marker. Staining was quantified using the semi-automated Opera Phenix high-content imager and the associated Columbus software, which uses light intensity thresholds to identify positive stains for each wavelength. Thresholds were determined using each plate’s reference serum wells and media control wells, allowing for a consistent approach in setting thresholds for staining batches with varying intensities while remaining blinded and unbiased.

### 2.5. Blood-Based Biomarkers

Plasma samples were analysed at the Department of Psychiatry and Neurochemistry, University of Gothenburg. Glial fibrillary acidic protein (GFAP) and neurofilament light chain (NFL) were quantified using the commercial Neurology 4-plex E kit (103670; Quanterix). Serum *p*-tau231 was analysed using in-house Simoa assays developed at the University of Gothenburg [24]. Serum *p*-tau217 was analysed with commercially available *p*-tau217 ALZpath Simoa *p*-tau217 V2 kits (104371; Quanterix), which use a proprietary monoclonal *p*-tau217-specific capture antibody, an *N*-terminal detector antibody, and a peptide calibrator (see Table S4 for average levels).

### 2.6. Statistical Analysis

All statistical analyses were conducted using R (version 4.4.0) prior to unblinding to group allocation. This study was carried out on a subset of participants with no missing data; therefore, it did not include an intention-to-treat analysis. Regression models were visually inspected using residuals vs. fitted values and QQ plots to assess normality and identify potential outliers. No influential outliers were identified, and no data points were excluded from the analyses. False discovery rate (FDR) correction was applied to account for multiple tests of variables of interest, resulting in q values reported in respective tables. When no q-value is reported, the variable was included in the model as a covariate rather than a variable of interest and, therefore, was not accounted for in the FDR. For further information on the statistical analysis, see Supplementary information. Analyses are briefly outlined below.

Beta regression models (glmmTMB package) were used to assess if oral ACN supplementation was associated with in vitro neurogenesis readouts before and after the intervention. When the models showed a significant interaction between intervention and visit (see Table 2), post hoc contrast tests were carried out to understand the effect size of these associations using the “emmeans” R package (version 1.10.4).

Linear mixed-effects models were used to investigate the effect of the intervention on hippocampal-dependent cognitive performance, which was measured by DPICOACC, DPICNACC, and CMBs.

Linear models were used to investigate whether baseline BBM levels were associated with neurogenesis readouts and whether neurogenesis readouts could predict cognitive outcomes at the endpoint, independent of the intervention. The R function “step” was used for forward selection based on the Akaike information criterion (AIC).

In the baseline analysis, the outcome variable for each model was the percentage of positive cells for each marker, and the explanatory variables were the BBM readouts and demographic and technical information. Similarly, for the longitudinal analysis to predict endpoint cognitive outcomes, stepwise forward selection using the “Step” function in R was used to identify significant variables. Individual models were built for each cognitive score, with starting models including baseline performance for the respective cognitive measure.

## 3. Results

### 3.1. ACN Intervention Impacts SOX2

HPCs treated with endpoint serum from the active intervention group showed significantly lower (0.0013%) levels of the stemness marker Sox2 compared to HPCs treated with respective baseline serum samples. Post hoc analysis confirmed that though modest (Cohen’s d = −0.20), this was significant (*p* = 0.03) and not present in the placebo group. Final covariates included in each model are reported in Table 2. Map2 levels appeared to be differentially affected by the placebo and active interventions; however, this did not survive the correction for multiple testing (Table S5 reports complete model outputs). The intervention did not have significant effects on the remaining neurogenesis markers.

### 3.2. ACN Intervention Does Not Impact Cognition

Given the change in neurogenesis markers, we investigated whether the intervention impacted performance on hippocampus-dependent cognitive tasks. This analysis differed from the trial’s primary outcome analysis as it focussed specifically on determining its impact on three cognitive readouts: DPIOACC, DPINACC, and their combined score (CMB). Our analysis revealed no significant effect of the intervention at the endpoint, though this may have been impacted by a learning effect (Table S6 and Figure S1). Covariates included in each final model are reported in Table 3, and full model outputs are reported in Table S7.

### 3.3. NFL GFAP pTAU at Baseline Are Associated with Neurogenesis Markers but Do Not Impact ACN Intervention Effects

Next, we focused on a smaller subset of participants with available data on both neurogenesis readouts and cognitive performance. This subset (*n* = 122) (Table S1) had available data on blood-based levels of key proteins related to central nervous system health (i.e., GFAP, *p*-tau217, *p*-tau231, and NFL).

We first established whether these readouts were associated with markers of the neurogenic process at baseline. The levels of several BBMs were correlated to markers of neurogenesis (Table 4). Of note, higher GFAP levels were associated with higher CC3p levels, and lower Ki67d and *p*-tau231 levels were positively associated with Ki67d. Several other potential correlations did not survive multiple testing correction (Table 4).

Finally, we repeated the beta regressions and mixed-effects linear models described in Section 3.1 and Section 3.2 in the smaller but more characterised subcohort. We included BBM levels (i.e., baseline and endpoint levels of *p*-tau217, *p*-tau231, GFAP, and NFL) in the stepwise selection process to assess if controlling for this data significantly altered our earlier conclusions. This analysis revealed similar effect sizes and direction to our original analysis, supporting our earlier conclusions (see Tables S8 and S9).

### 3.4. Markers of Apoptotic Cell Death Are Significantly Associated with Hippocampal-Dependent Cognitive Performance

We then sought to establish the in vivo implications of altered in vitro neurogenesis markers again using the subcohort described in Table S1. We tested if variation in our neurogenesis markers, following treatment with participant serum, predicts changes in the respective participant’s cognitive performance. We employed linear models to predict endpoint cognitive performance in each of the three cognitive tasks based on changes in neurogenesis and BMM marker levels from baseline to the endpoint. Apoptosis levels, measured via CC3 levels, were positively associated with performance in all three cognitive measures (Table 5), suggesting that increased apoptosis is associated with improved endpoint cognitive performance. We also report risk-type diagnosis, BMI, ApoE4 carrier status, and GFAP levels as important negative determinants of cognitive performance.

### 3.5. BMI and ApoE Have a Moderator Effect on Neurogenesis in Cognition

Finally, given the role of BMI and ApoE4 carrier status in dementia and cognitive decline, we assessed whether these acted as moderators for the association of neurogenesis on cognitive performance by including interactions between neurogenesis readouts and BMI or ApoE4 carrier status.

**Figure 2:**
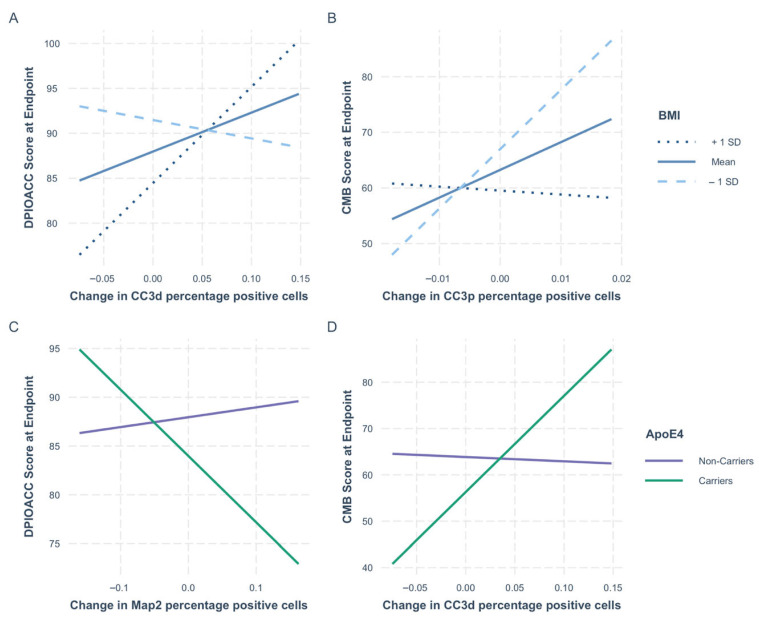
Moderation effect of BMI and ApoE4. Graphical representation of the interaction results reported in Table 5. Top row shows the impact of BMI on (**A**) the association between the change in CC3d and original Cue accuracy performance at the endpoint (DPIOACC) and (**B**) the change in CC3p and original and novel cure accuracy combined scores at the endpoint (CMB). BMI was treated as a continuous variable in this analysis; for ease of representation and to understand the impact of varying BMIs, we show the average association between the marker and the outcome for mean BMI (BMI = 28.4, dashed line), mean BMI +1SD (BMI = 32.7, solid line), and mean BMI -1SD (BMI = 24.2, dotted line). Bottom row shows the impact of ApoE4 carrier status. (**C**) shows how the association between the change in Map2 and performance on the original cue accuracy task (DPIOACC) is moderated by carrier status, while (**D**) shows how the association between the change in CC3d and the original and novel combined scores (CMB) is moderated. For this analysis, ApoE4 was treated as a categorical variable; associations for carriers for one or two ApoE4 alleles are shown in green, while those for non-carriers are shown in purple. CC3p stands for the percentage of hippocampal progenitor cells positive for the CC3 marker following the proliferation assay. CC3d stands for the percentage of hippocampal progenitor cells positive for the CC3 marker following the differentiation assay. The Map2 readout is the percentage of hippocampal progenitor cells positive for the Map2 marker following the differentiation assay. Each participant’s change in each marker is calculated by subtracting the percentage of positive cells for the respective marker following treatment with serum samples collected at the endpoint minus the percentage of positive cells for that marker following treatment with serum samples collected at baseline visits.

Firstly, we found that BMI moderates the association of CC3p and CC3d with cognitive performance (Figure 2 and Table 5). BMI moderates the effect of apoptosis during differentiation (CC3d) on DPIOACC performance (*p* = 0.02); CC3d is negatively associated with cognition in participants with healthy BMIs (*p* = 0.045) but positively associated in participants with BMIs in the obese range (*p* = 0.02) (Table S10). In addition, BMI impacted the association between apoptosis in proliferation (CC3p) and CMB performance (*p* = 0.03); CC3p is only associated with cognitive performance in individuals with healthy BMIs (*p* = 0.05) (Table S11).

Further, ApoE4 carrier status moderated the association between Map2 and DPIOACC performance (*p* = 0.008) and had a potential moderator effect on CC3p and CMB performance (*p* = 0.04) (Figure 2 and Table 5). While there is no overall association between Map2 levels and cognition (*p* = 0.65), stratification according to ApoE4 status showed a negative association between Map2 and DPIOACC performance in ApoE4 carriers (*p* = 0.01) and no association in non-carriers (*p* = 0.55) (Table S12). Similarly, in ApoE4 carriers there was a positive association between CC3d and cognitive performance (*p* = 0.01), while no association was present in non-carriers (*p* = 0.99) (Table S13).

## 4. Discussion

The ACID trial is the largest and most comprehensive ACN trial to date and was designed to test if ACNs could affect relevant mechanisms linked to cognitive decline and dementia. Here, we show that ACN supplementation is able to impact in vitro neurogenesis and that this can impact hippocampal-dependent cognition. We show for the first time that blood-based dementia biomarkers correlate with human in vitro neurogenesis markers and that these markers can help predict cognitive performance. We also highlight that key demographic characteristics such as BMI and ApoE4 carrier status can moderate the effect of neurogenesis on cognition, supporting the need for more individualised trials.

### 4.1. Decrease in Stem Cell Integrity Following Oral ACN Supplementation

Treatment of HPCs with serum obtained following the active intervention caused a 0.0013% decrease in the HPC percentage of HPCs positive for Sox2, a transcription factor responsible for transcriptional response regulation to neurogenic cues. This suggests that anthocyanins can alter the systemic environment in a way that can impact HPC regulation. A decrease in Sox2 is associated with a decrease in progenitor integrity and a push towards differentiation [25] and has been linked to a shift in radial glial morphotypes [26]. Within our controlled system, the HPCs are kept in a proliferative state by the presence of growth factors and 4OHT, which may mask the greater effect of ACN-supplemented serum on HPCs.

### 4.2. Intervention and Hippocampal Cognition

We previously reported that the ACID intervention did not affect overall cognition, which was measured as a composite score of cognitive performance across different domains [21]. This secondary study focuses on ACN’s effect on neurogenesis and hippocampus-dependent cognitive tasks: original object recognition (DPIOACC), novel object recognition (DPINACC), and their composite measure CMB. Though an improvement in cognitive performance in an at-risk population was unlikely, we hypothesised that a dietary intervention could slow or prevent cognitive decline. However, the cognitive trajectory of participants did not differ across intervention arms. To our knowledge, ACID is the longest ACN intervention trial to date; still, a longer intervention or follow-up may be needed to identify such cognitive effects, especially given the promising impact on the neurogenesis markers. ACID benefitted from rich data availability and repeated monthly measures using a fully online cognitive battery [14]. However, a potential learning effect may have masked intervention effects, highlighting the need to retest the presence of learning effects across commonly used batteries as trials move towards remote and, therefore, often more frequent time points.

### 4.3. Blood-Based Biomarkers Are Associated with the Neurogenic Process at Baseline

The ACID cohort is the only currently available human cohort with data on AD BBMs and neurogenesis readouts; we thus investigated how these two measures, both strongly associated with dementia, correlated to one another. We found that levels of GFAP, *p*-tau217, *p*-tau231, and NFL each correlated with at least one measure of neurogenesis at baseline.

Higher levels of GFAP, a brain-specific cytoskeletal intermediate filament protein found in astrocytes, predicted increased apoptosis levels during the proliferation assay (CC3p) and lower proliferation (Ki67d). This aligns with the existing literature showing that GFAP levels are associated with AD, cognitive decline, and amyloid beta plaques, which are all thought to be associated with lower neurogenesis levels [27,28]. As blood-based GFAP levels are often used as a proxy for reactive astrocyte levels in vivo and thereby as a measure of neuroinflammation, our data supports the notion that higher neuroinflammation is associated with decreased neurogenesis [29].

In contrast, higher pTau213 levels were linked to increased proliferation (higher Ki67 expression during differentiation), and *p*-tau217 levels showed similar trends with associations to a higher number of immature neurons (Map2) and decreased neural progenitor integrity (Sox2 and Nestin). Phosphorylated tau (pTau) is amongst the leading BBMs for biofluid-based dementia detection, as tau phosphorylation is a key hallmark of AD pathology. Studies have shown that *p*-tau217 and *p*-tau217, i.e., pTau phosphorylated at threonine 231 and threonine 217, can reliably detect biological AD [24,30]. Here pTau is associated with a push towards creating new neurons. In contrast, GFAP appears to be linked to the opposite, suggesting that these markers may relate to different disease stages. Though no participant has an existing dementia diagnosis, part of the cohort was likely in the prodromal stages of pathology. This is corroborated by the *p*-tau217 levels ranging from 0.130 pg/mL to 1.39 pg/mL in our cohort and the current *p*-tau217 cutoff for biological dementia being >0.63 pg/mL [30].

Similarly, though non-significant, increased NFL levels may be associated with higher neuroblast, immature neuron, and proliferation levels. Suggesting that neuronal damage, of which NFL levels are a proxy, boosts neurogenesis as an endogenous repair mechanism [31]. This trial was not designed to assess the association between BBMs and neurogenesis markers. Therefore, we are limited regarding the in vivo relevance and conclusions we can draw from this data. Nonetheless, the associations between BBMs and neurogenesis support that HPCs may mediate the effects of known dementia BBMs. Further research on this association will help devise more individualised therapies and enrich trials with participants with specific biomarker profiles most likely to benefit from certain interventions.

### 4.4. Neurogenesis Markers and AD Blood-Based Biomarkers Can Predict Cognitive Performance

Though the intervention did not impact cognition, it impacted neurogenesis, so understanding how these factors are associated could give us insight into the potential future relevance of the intervention. In this cohort, the serum samples used in the neurogenesis assay were collected at the same time as the cognitive measures, so it is possible that this design does not measure any downstream effects of neurogenesis changes on cognition (e.g., the decrease in Sox2), as it takes several weeks for the process of neurogenesis to be fully complete and to begin impacting behaviour. Still, we show that increased apoptosis during the proliferation assay was associated with better hippocampal-dependent performance.

We also show that apoptosis is linked to better performance in original object recognition in participants with low BMIs, while the opposite was true for participants with high BMIs. This suggests a potential shift from beneficial and controlled apoptosis in lower BMIs, which is needed for efficient neurogenesis, to less beneficial and perhaps more untargeted apoptosis in higher BMIs. In line with this, apoptosis was only associated with better composite CMB performance in lower BMIs.

Similarly, ApoE4 carriers showed a positive association between apoptosis and CMBs and negative associations between the number of immature neurons and the original accuracy scores. In contrast, non-carriers showed no association with either.

As ApoE4 alleles and BMI are both thought to increase the risk of dementia, the apoptosis results at first appear surprising. Still, a similar interaction was found in environmental enrichment experiments comparing ApoE4 and ApoE3 mice [32], supporting further research to elucidate the relationship between apoptosis and neurogenesis in ApoE4 genotypes. Though further cohort studies explicitly aimed at testing the moderator effect of these risk factors are needed to draw robust conclusions, our results suggest that the impact of BMI and ApoE4 on dementia risk may be mediated via their effects on HPCs, further supporting the need for more individualised clinical trials.

Finally, we report on a secondary analysis of an existing cohort; the study design limitations include an intervention duration of only 6 months rather than lifetime exposure and the inclusion of a single dose, preventing dosage effect analysis. Furthermore, the lack of food diaries prevented us from accounting for potential changes in diet throughout the intervention and for baseline intake levels of anthocyanins, which, in a Norwegian population, may be higher than average [33,34]. Previous work has also failed to detect a convincing increase in anthocyanin metabolites following this intervention, suggesting the potential of a ceiling effect or a lack of efficient absorption [15]. Despite this, the ACID trial remains one of the most comprehensive ACN trials to date and allowed for initial proof-of-concept analysis, linking human neurogenesis, BBMs, and cognition for the first time.

## 5. Conclusions

In conclusion, we show that dietary oral supplementation can alter the composition of the systemic environment, which can affect HPC regulation. Our data supports a role for neurogenesis in cognitive performance and shows that participant characteristics such as BMI and ApoE4 carrier status can moderate these relationships. Finally, our findings support further research into the mechanism of action of ACNs and the use of BBMs for clinical trial enrichment, trial individualization, and therapy development.

## Figures and Tables

**Table 1:** Cohort characteristics.

	Active (*N* = 90)	Placebo (*N* = 91)	Overall (*N* = 181)
Age			
Mean (SD)	69.2 (5.25)	69.5 (5.60)	69.4 (5.41)
Median [Min, Max]	68.0 [60.0, 79.0]	69.0 [60.0, 80.0]	69.0 [60.0, 80.0]
Sex			
Female	45 (50.0%)	45 (49.5%)	90 (49.7%)
Male	45 (50.0%)	46 (50.5%)	91 (50.3%)
Education			
Mean (SD)	14.6 (3.42)	13.8 (2.97)	14.2 (3.22)
Median [Min, Max]	15.0 [8.00, 22.0]	14.0 [8.00, 20.0]	14.0 [8.00, 22.0]
ApoE			
E4 non-carrier	54 (60.0%)	54 (59.3%)	108 (59.7%)
E4 carrier	36 (40.0%)	37 (40.7%)	73 (40.3%)
Risk			
CMD	64 (71.1%)	58 (63.7%)	122 (67.4%)
MCI	26 (28.9%)	33 (36.3%)	59 (32.6%)
BMI			
Mean (SD)	27.3 (3.86)	28.3 (4.61)	27.8 (4.28)
Median [Min, Max]	26.9 [19.9, 38.4]	28.7 [19.6, 41.4]	27.7 [19.6, 41.4]
Site			
Stavanger	48 (53.3%)	51 (56.0%)	99 (54.7%)
Bergen	30 (33.3%)	31 (34.1%)	61 (33.7%)
Akershus	12 (13.3%)	9 (9.9%)	21 (11.6%)

Cohort characteristics for the subset of ACID participants with available serum samples. Education was measured in years. ApoE refers to ApoE4 carrier status. Risk refers to whether participants were considered at risk for dementia due to a cardiometabolic disorder (CMD) or a mild cognitive impairment (MCI) diagnosis.

**Table 2:** Intervention effect on neurogenesis markers.

Outcome	Intervention * Visit			Covariates
	Estimate	*p*	q	
**Sox2**	**−0.42**	**<0.0001**	**0.0008**	**BMI, Education, ApoE, Plate Location, Test Site, Staining Batch**
Nestin	−0.04	0.30	0.53	BMI, MCI, Plate Location, and Staining Batch
Ki67 proliferation	0.02	0.53	0.71	Staining Batch and Test Site
CC3 proliferation	−0.005	0.86	0.93	Staining Batch, Plate Location, and Test Site
Ki67 differentiation	−0.039	0.24	0.53	Staining Batch, Risk Type, and Test Site
DCX	0.008	0.93	0.93	Staining Batch and Test Site
Map2	−0.13	0.04	0.16	Staining Batch, Plate Location, and Test Site
CC3 differentiation	0.0428	0.33	0.53	Age, Staining Batch, and Test Site

Table showing results for each model investigating the effect of the intervention on each marker of the neurogenic process. Respective beta estimates, *p*-values, q-values following the FDR and covariates are reported for each model. Significance (q < 0.05) is shown in bold. “*” represents an interaction between two terms.

**Table 3:** Intervention effects on hippocampus-dependent cognition.

Outcome	Intervention * Visit			Covariates
	Chisq	*p*	q	
DPIOACC	0.19	0.66	0.20	Age, Education, Risk Type, and ApoE
DPINACC	0.73	0.39	0.59	Age and Test Site
CMB	0.10	0.75	0.75	Age, Education, MCI, and ApoE

Results of each linear mixed-effects model aimed at determining the effect of the intervention on DPIOACC, DPINACC, and CMB. Respective beta estimates, *p*-values, q-values following the FDR and covariates are reported for each model. “*” represents an interaction between two terms.

**Table 4:** Effect of blood-based biomarkers of dementia on neurogenesis markers.

	Sox2 Adj R = 0.05 F = 3 *p* = 0.03			Nestin Adj R = 0.14 F = 5 *p* = 0.0003			CC3p Adj R = 0.19 F = 10 *p* ≤ 0.0001			Ki67p Adj R = 0.08 F = 12 *p* = 0.0006			DCX Adj R = 0.45 F = 26 *p* < 0.0001			CC3d Adj R = 0.65 F = 115 *p* ≤ 0.0001			Ki67d Adj R = 0.19 F = 6 *p* ≤ 0.0001			Map2 Adj R = 0.40 F = 16 *p* < 0.0001		
	β	*p*	q	β	*p*	q	β	*p*	q	β	*p*	q	β	*p*	q	β	*p*	q	β	*p*	q	β	*p*	q
BMI	-	-	-	0.0004	0.04	-	-	-	-	-	-	-	-	-	-	-	-	-	-	-	-	-	-	-
Risk Type	0.009	0.03	-	-	-	-	-	-	-	-	-	-	-	-	-	-	-	-	0.013	0.15	-	-	-	-
Age	-	-	-	-	-	-	-	-	-	-	-	-	-	-	-	-	-	-	0.002	0.057	-	-	-	-
ApoE4 status	-	-	-	-	-	-	−0.004	0.04	-	-	-	-	-	-	-	0.019	0.08	-	-	-	-	-	-	-
Staining batch	−0.001	0.10		-	-	-	-	-	-	0.005	0.0006	-	0.013	<0.0001		−0.06	<0.0001	-	0.009	0.005	-	−0.07	<0.0001	-
Plate location	-	-	-	0.002	0.006		-	-	-	-	-	-	0.003	0.08		-	-	-	-	-	-	-	-	-
Test site	-	-	-	-	-	-	−0.005	0.003		-	-	-	0.01	0.03		-	-	-	-	-	-	−0.04	0.10	-
**GFAP**	-	-	-	-	-	-	**0.00008**	**<0.0001**	**0.001**	-	-	-	-	-	-	-	-	-	**−0.0002**	**0.0089**	**0.030**	−0.0007	0.03	0.067
*p*-tau217	−0.012	0.06	0.067	−0.004	0.06	0.067	-	-	-	-	-	-	-	-	-	-	-	-	-	-	-	0.12	0.04	0.067
***p*** **-tau231**	-	-	-	-	-	-	-	-	-	-	-	-	-	-	-	-	-	-	**0.002**	**0.0006**	**0.003**	-	-	-
NFL	-	-	-	-	-	-	-	-	-	-	-	-	0.0005	0.06	0.067	-	-	-	0.001	0.08	0.080	0.004	0.06	0.067

Model fit for each marker of the neurogenic process as the outcome measure (column headings). The estimates and *p*-values for each variable in the final model are reported in each row. FDR correction was applied to account for all variables of interest in the final models, and q-values following the FDR are reported. Significance (q < 0.05) is shown in bold.

**Table 5:** Effect of neurogenesis markers, BMI, and ApoE carrier status on hippocampus-dependent cognition.

	DPIOACC Adj R = 0.49, F statistic = 14, *p* < 0.0001			DPINACC Adj R = 0.43, F statistic = 27, *p* < 0.0001			CMB Adj R = 0.51, F statistic = 13, *p* < 0.0001		
	β	*p*	q	β	*p*	q	β	*p*	q
BL cognition	0.56	<0.0001	-	0.62	<0.0001	-	0.64	<0.0001	-
ApoE	−76,520	0.037	-	-	-	-	−7.55	0.046	-
BMI	−17,230	0.0002	-	-	-	-	−0.87	0.06	-
Risk Type	−131,600	0.003	-	-	-	-	−6.86	0.12	-
Map2	166,300	0.65	0.72	-	-	-	-	-	-
CC3d	−8,810,000	0.045	0.061	-	-	-	−9.29	0.91	0.91
**CC3p**	-	-	-	**466.70**	**0.01**	**0.046**	**4288.24**	**0.01**	**0.046**
**GFAP**	-	-	-	**−0.08**	**0.02**	**0.046**	−0.09	0.06	0.07
CC3d * BMI	**340** **,** **800**	**0.02**	**0.046**	-	-	-	-	-	-
ApoE * Map2	**−1** **,** **560** **,** **000**	**0.008**	**0.046**	-	-	-	-	-	-
CC3p * BMI	-	-	-	-	-	-	**−133.36**	**0.03**	**0.046**
ApoE * CC3d	-	-	-	-	-	-	216.98	0.04	0.06

Linear model results of each model (column headers), the model fit (column headers), the estimates, and *p*-values of each variable included in the final model for models predicting cognitive performance. For this analysis, full models included potential interactions between ApoE4 status/BMI and neurogenesis markers. DPICOACC values are cubed. FDR correction was applied to account for all variables of interest in the final models, and the q-values following the FDR are reported. Significance (q < 0.05) is shown in bold.“*” represents an interaction between two terms.

## Data Availability

The datasets generated and/or analysed during the current study are not publicly available due ethics restrictions; some data are available from the corresponding author upon reasonable request.

## Supplementary Materials

The following are available online at https://www.mdpi.com/article/10.3390/nu17162680/s1. Table S1: Sub-cohort characteristics—Subcohort included the subset of ACID participants with available serum samples, cognitive measures and blood-based biomarker data. Education was measured in years. ApoE refers to ApoE4 carrier status. Risk refers to whether participants were considered at risk for dementia due to a cardiometabolic disorder (CMD) or a mild cognitive impairment (MCI) diagnosis. Table S2: Immunocytochemistry details- Table showing the concentrations and catalog numbers of the antibodies used for immunocytochemistry. Table S3: Neurogenesis markers at baseline and endpoint—Table showing baseline(BL) and endpoint (EP) values for each neurogenesis marker in each intervention group. Table S4: Blood-based biomarkers at baseline and endpoint—Table showing baseline (BL) and endpoint (EP) values of blood-based biomarkers. Table S5: Beta regression results for full cohort—Tables reporting full results for each Beta-regression model aimed at determining the effect of the intervention on neurogenesis markers. Table S6: Summary of cognitive performance trajectories—For the purposes of this table only, participants were classed as stable if their scores remained within a 10 point range across the two timepoints (baseline and endpoint). Alternatively they were classed as “improved” or “declined” if their scores improved or declined by at least 5 points from baseline to endpoint. Table S7: Full model outputs of linear mixed effect models—Results of each linear mixed effect model aimed at determining the effect of the intervention on DPIOACC, DPINACC and CMB. Full model outputs relating to Table 3. Table S8: Beta regression results for sub-cohort—Tables reporting full results for each Beta-regression model aimed at determining the effect of the intervention on neurogenesis markers in sub cohort of participants with available BBM information. Table S9: Mixed effect models in sub-cohort—Results of each linear mixed effect model aimed at determining the effect of the intervention on DPIOACC, DPINACC and CMB in sub-cohort of participants with available BBM information. Table S10: Results of stratification analysis investigating the moderator effect of BMI on DPICOACC performance—Left shows linear model results when analysis was restricted to individuals who were in the “healthy” BMI range (BMI of 18.5–24.9, n = 25). Middle shows results of the analysis restricted to individuals within the “overweight” range (BMI of 25–29.9, n = 42) and on the right are the results of the analysis restricted to individuals in the “obese” BMI range (BMI >29.9, n =37). The estimates and p values of each variable originally included in the final model for predicting DPICOACC performance. Results show opposite CC3d associations with cognitive performance in the lowest and highest BMI groups. DPICOACC values are cubed. Table S11: Results of stratification analysis investigating the moderator effect of BMI on CMB performance—Left shows linear model results when analysis was restricted to individuals who were in the “healthy” BMI range (BMI of 18.5–24.9, n = 25). Middle shows results of the analysis restricted to individuals within the “overweight” range (BMI of 25–29.9, n = 42) and on the right are the results of the analysis restricted to individuals in the “obese” BMI range (BMI >29.9, n =37). The estimates and p values of each variable originally included in the final model for predicting CMB performance. Results show CC3p is associated with cognitive performance in only the healthy BMI group. Table S12: Results of stratification analysis investigating the moderator effect of ApoE4 status on CMB performance—Left shows linear model results when analysis was restricted individuals who were non-carriers for ApoE4 (n = 62). Right shows results of analysis in the subset of participants who carried one or two E4 alleles (n =42). The estimates and p values of each variable originally included in the final model for predicting DPICOACC performance. DPICOACC values are cubed. Results show Map2 is only associated with cognitive performance in ApoE4 carriers. Table S13: Results of stratification analysis investigating the moderator effect of ApoE4 status on DPICOACC performance—Left shows linear model results when analysis was restricted to individuals who were non-carriers for ApoE4 (n = 62). Right shows results of the analysis restricted to individuals who carried one or two E4 alleles (n =42). The estimates and p values of each variable originally included in the final model for predicting CMB performance. Results show CC3d is only associated with cognitive performance in ApoE4 carriers. Figure S1: Change in cognitive performance throughout ACID trial—Bar graphs showing the change in each cognitive measure: original cue accuracy (DPICOACC), novel cue accuracy (DPICNACC) and original and novel combination score (CMB) across both intervention arms. A positive value indicates improvement from baseline to endpoint. A negative value indicates decline.

## Abbreviations

The following abbreviations are used in this manuscript:

ACID	AnthoCyanins In people at risk for Dementia
ACNs	Anthocyanins
AHN	Adult Hippocampal Neurogenesis
AIC	Akaike Information Criterion
BBMs	Blood-Based Biomarkers
CMB	Cognitive Combination Score
DPICNACC	Picture Recognition New Stimuli Accuracy
DPICOACC	Picture Recognition Original Stimuli Accuracy
FDR	False Discovery Rate
GFAP	Glial Fibrillary Acidic Protein
HPCs	Hippocampal Progenitor Cells
NFL	Neurofilament Light Chain
NSCs	Neural Stem Cells
