# Supplementary material for: Effects of Oral Anthocyanin Supplementation on In Vitro Neurogenesis, Hippocampus-Dependent Cognition, and Blood-Based Dementia Biomarkers: Results from a 24-Week Randomized Controlled Trial in Older Adults At Risk for Dementia (ACID)
Source: Nutrients. 2025 Aug 19;17(16):2680. doi: 10.3390/nu17162680 (PMC12389521; doi:10.3390/nu17162680)
Supplement: Supplementary file 1 [file nutrients-17-02680-s001.zip › nutrients-3778509-supplementary.pdf]

Supplementary Materials

Methods

Table S1 *– Sub-cohort characteristics*

Participants

	Active (N=49)	Placebo (N=55)	Overall (N=104)
**Age**			
Mean (SD)	69.4 (4.82)	69.1 (5.52)	69.3 (5.18)
Median [Min, Max]	68.0 [60.0, 79.0]	69.0 [60.0, 80.0]	68.5 [60.0, 80.0]
**Sex**			
Female	26 (53.1%)	28 (50.9%)	54 (51.9%)
Male	23 (46.9%)	27 (49.1%)	50 (48.1%)
**Education**			
Mean (SD)	14.8 (3.50)	13.4 (2.87)	14.1 (3.25)
Median [Min, Max]	15.0 [8.00, 22.0]	13.0 [8.00, 18.0]	14.0 [8.00, 22.0]
**ApoE**			
e4 non-carrier	31 (63.3%)	31 (56.4%)	62 (59.6%)
e4 carrier	18 (36.7%)	24 (43.6%)	42 (40.4%)
**Risk**			
CMD	36 (73.5%)	37 (67.3%)	73 (70.2%)
MCI	13 (26.5%)	18 (32.7%)	31 (29.8%)
**BMI**			
Mean (SD)	28.1 (4.30)	28.7 (4.25)	28.4 (4.26)
Median [Min, Max]	28.3 [20.1, 38.4]	29.0 [19.6, 39.2]	28.6 [19.6, 39.2]
**Site**			
Stavanger	33 (67.3%)	31 (56.4%)	64 (61.5%)
Bergen	15 (30.6%)	21 (38.2%)	36 (34.6%)
Akershus	1 (2.0%)	3 (5.5%)	4 (3.8%)

Subcohort included the subset of ACID participants with available serum samples, cognitive measures and blood-based biomarker data. Education was measured in years. ApoE refers to ApoE4 carrier status. Risk refers to whether participants were considered at risk for dementia due to a cardiometabolic disorder (CMD) or a mild cognitive impairment (MCI) diagnosis.

Serum sample collection and storage

Serum samples for each participant were collected at baseline and endpoint visits. Participants were instructed to fast prior to the blood draw. Serum was separated from plasma via centrifugation at 1200G and stored at -75° C at each trial centre before being anonymised and shipped to King's College London on dry ice. There, it was aliquoted and stored at -75° C until needed in the neurogenesis assay. All samples used in this study had undergone one freeze-thaw cycle.

Cell culture

HPCs were grown on tissue culture flasks (Nunclon) freshly coated with 20μg/ml mouse laminin (L2020, Sigma). The media components used in this study varied slightly from the previously reported methodology and are reported below. Here, HPCs were grown in reduced modified media (RMM) consisting of Dulbecco's Modified Eagle's Media/F12 (21331020, Gibco) supplemented with 15nM HEPES (5630080, Gibco), 0.03% human albumin (Albunorm 20%, Octapharma, UK), 100 μg/ml human apo-transferrin (T1147, Sigma), 16.2 μg/ml human putrescine DiHCl (P5780, Sigma), 5 μg/ml human recombinant insulin (I9278, Sigma), 60ng/ml progesterone (P8783, Sigma), 2 mM L-glutamine (G7513, Sigma) and 40 ng/ml sodium selenite (S9133, Sigma). During passaging and during the proliferation assay the above media was further supplemented with 10 ng/ml human bFGF (EC100-18B, Peprotech), 20 ng/ml human EGF (AF100-15-500, Peprotech) and 100 nM 4-OHT (H7904, Sigma). All experiments were carried out on passage numbers 20-25.

Proliferation and differentiation assays

For experiments, HPCs were seeded at a density of $1.2X10^4$ in 96-well plates (Nunclon) and treated 24 hours after seeding. HPCs undergoing the proliferation assay were left in culture for 48 hours, then fixed in 4% PFA for 20 minutes and stored at 4° C in PBS and NaN3. Cells destined for the differentiation assay instead were also left to proliferate for 48 hours since treatment. They were then washed in RMM, treated again with respective serum samples, and left to differentiate for 7 days. Following this, they were fixed and stored as described above. Treatment consisted of 1% participant serum diluted in either proliferation media (24-hour timepoint) or RMM media for treatment during the differentiation assay. After serum treatment, media was supplemented with 0.5 mg/ml Penicillin Streptomycin (10,000 U/ml, 15140-122, Life Technologies). Baseline and endpoint samples for each participant were always run on the same plate, and reference serum and penicillin-media controls were present on each plate.

Table S2 *– Immunocytochemistry details*

	Provider	Cat no	Dilution
**Primaries**			
Rabbit Sox2	Chemicon	ab5603	1 in 1000
Mouse Nestin	Chemicon	mab5326	1 in 1000
Rabbit Cleaved Caspase3	CellSignalling	#9664	1 in 500
Mouse ki67	CellSignalling	9449	1 in 800
Rabbit Dcx	Abcam	ab18723	1 in 500
Mouse Map2	Abcam	ab11267	1 in 500
**Secondaries**			
Donkey anti-mouse 488	Life technologies	A21202	1 in 500
Donkeyanti-rabbit 555	Life technologies	A31572	1 in 500

Table showing the concentrations and catalog numbers of the antibodies used for immunocytochemistry.

Cognitive tasks

During the encoding phase, participants are shown 20 pictures at the rate of 1 every 3 seconds. After 15 minutes, the original 20 pictures are presented together with 20 similar pictures. Participants are asked to distinguish between original and novel, similar images. The DPICOACC score is then calculated based on the percentage of correctly identified original pictures, whereas the DPICNACC score is calculated based on the percentage of correctly identified novel pictures. The CMB score is calculated by adding the DPICOACC and DPICOACC scores and subtracting 100. Participants are shown different picture pairs at each visit to minimize learning effects. A small subset of participants underwent delayed cognitive assessment at the endpoint, we only selected participants who had undergone their last cognitive assessments by week 26.

Statistical analysis

Variable coding and rescaling

ApoE status was defined as a binary variable for all analyses, with "0" assigned to non-carriers and "1" to heterozygous or homozygous ApoE-ε4 carriers. Education was defined as years of education, and sex was treated as a binary variable. Risk type was coded as a binary variable, with "0" assigned to participants with cardiometabolic risk and "1" to those with MCI. The test centre was a categorical variable treated as a dummy.

Additionally, staining batch refers to the batch ID in immunocytochemistry staining, and plate location refers to the column within the 96-well plate where each participant's serum was located. The percentage of positive cells for each ICC marker was re-coded as a value between 0 and 1. Three markers were rescaled: SOX2 ((1-x)10), Nestin (1-x), and DCX (x0.2) to improve model efficiency. The rescaled variables were used for all analyses involving these three markers.

SOX2 showed values close to or equal to 100% in several samples across both time points; this translated to a high proportion of zero values in the transformed variable. A two-part model was employed in beta regression models predicting SOX2, where one part modelled the probability of a zero result.

Beta regressions

Independent models were used to predict the intervention effect on each marker (Sox2, Nestin, Ki67p, CC3p, Ki67d, Map2, DCX, CC3d). Backwards stepwise regression was employed to define the final models using a covariate inclusion threshold of p<0.1. Full models included the explanatory variables: "intervention" and "visit" and their interaction; the following clinical variables: sex, age, BMI, education, test centre, risk type, and ApoE4 status; and the technical variables: staining batch and location in the plate. Beta regressions were repeated as above in the sub-cohort with BBM data. For these, full models prior to backwards selection included the variables above as well as p-tau217, p-tau231, GFAP and NFL.

Mixed-effect linear models

Backward stepwise regression was applied to identify significant covariates, with a threshold for covariate inclusion set at $p<0.01$. Full models included the following covariates: sex, age, BMI, education, MCI diagnosis, test centre, and ApoE4 carrier status. Random intercepts based on participant ID were included in the model. The explanatory variables of interest were "visit", "intervention", and their interaction. The "DPICOACC" scores were cubed-transformed to satisfy the normality assumption. The transformation was applied to baseline and endpoint scores and was retained in all subsequent analyses of this outcome. Linear mixed-effect models were repeated as above in the sub-cohort with BBM data. For these, full models prior to backwards selection included the variables above as well as p-tau217, p-tau231, GFAP and NFL.

Linear regressions

In the baseline analysis testing whether baseline BBM levels were associated with neurogenesis readouts, the variables tested for inclusion were p-tau217, p-tau231, GFAP, NFL, sex, age, BMI, education, test site, ApoE4 carrier status and MCI diagnosis. The longitudinal analysis, aimed to predict vognitive outcomes, here the potential full models included change in SOX2, Nestin, Ki67p, CC3p, Ki67d, DCX, MAP2, CC3d, p-tau217, p-tau231, GFAP, NFL, as well as sex, age, BMI, education, test site, ApoE4 carrier status, MCI diagnosis, and intervention arm. VIF values over five were assumed to indicate potential collinearity, and no values exceeded this threshold. For linear models aimed at determining whether there was a modulatory effect of BMI or ApoE4 carrier status, the above methodology was used, but

interaction terms between each neurogenesis readout and BMI/ApoE4 status were included in the full models.

Results

Table S3 - *Neurogenesis markers at baseline and endpoint.*

	Active		Placebo		Overall	
	**BL (N=90)**	**EP (N=90)**	**BL (N=91)**	**EP (N=91)**	**BL (N=181)**	**EP (N=181)**
**Ki67p**						
Mean (SD)	0.875 (0.0360)	0.877 (0.0298)	0.872 (0.0349)	0.877 (0.0325)	0.874 (0.0354)	0.877 (0.0311)
Median [Min, Max]	0.884 [0.747, 0.926]	0.882 [0.777, 0.920]	0.884 [0.763, 0.926]	0.885 [0.789, 0.934]	0.884 [0.747, 0.926]	0.883 [0.777, 0.934]
**CC3p**						
Mean (SD)	0.0291 (0.0129)	0.0301 (0.0148)	0.0302 (0.0121)	0.0311 (0.0130)	0.0297 (0.0125)	0.0306 (0.0139)
Median [Min, Max]	0.0275 [0.00949, 0.0649]	0.0269 [0.00946, 0.0705]	0.0297 [0.00945, 0.0603]	0.0287 [0.00994, 0.0659]	0.0290 [0.00945, 0.0649]	0.0280 [0.00946, 0.0705]
**SOX2**						
Mean (SD)	0.999 (0.00214)	0.999 (0.00291)	0.999 (0.00169)	0.999 (0.00157)	0.999 (0.00192)	0.999 (0.00233)
Median [Min, Max]	1.00 [0.982, 1.00]	1.00 [0.975, 1.00]	1.00 [0.991, 1.00]	1.00 [0.992, 1.00]	1.00 [0.982, 1.00]	1.00 [0.975, 1.00]
**Nestin**						
Mean (SD)	0.985 (0.00667)	0.984 (0.00953)	0.984 (0.00785)	0.984 (0.00585)	0.984 (0.00727)	0.984 (0.00788)
Median [Min, Max]	0.985 [0.934, 0.993]	0.985 [0.915, 0.993]	0.985 [0.929, 0.993]	0.985 [0.955, 0.993]	0.985 [0.929, 0.993]	0.985 [0.915, 0.993]
**Ki67d**						
Mean (SD)	0.214 (0.0525)	0.224 (0.0524)	0.217 (0.0458)	0.222 (0.0520)	0.216 (0.0492)	0.223 (0.0520)
Median [Min, Max]	0.213 [0.0915, 0.330]	0.218 [0.117, 0.374]	0.220 [0.0765, 0.331]	0.218 [0.112, 0.362]	0.216 [0.0765, 0.331]	0.218 [0.112, 0.374]
**DCX**						
Mean (SD)	0.507 (0.180)	0.534 (0.179)	0.510 (0.176)	0.533 (0.198)	0.509 (0.177)	0.534 (0.188)
Median [Min, Max]	0.475 [0.150, 0.809]	0.509 [0.138, 0.982]	0.494 [0.177, 0.803]	0.493 [0.154, 0.994]	0.481 [0.150, 0.809]	0.501 [0.138, 0.994]
**CC3d**						
Mean (SD)	0.123 (0.0963)	0.121 (0.0897)	0.123 (0.101)	0.126 (0.101)	0.123 (0.0985)	0.124 (0.0951)
Median [Min, Max]	0.0797 [0.0211, 0.420]	0.0867 [0.0231, 0.366]	0.0787 [0.0192, 0.441]	0.0864 [0.0177, 0.433]	0.0796 [0.0192, 0.441]	0.0864 [0.0177, 0.433]
**MAP2**						
Mean (SD)	0.719 (0.179)	0.733 (0.182)	0.730 (0.158)	0.722 (0.166)	0.725 (0.168)	0.727 (0.174)
Median [Min, Max]	0.800 [0.183, 0.939]	0.819 [0.230, 0.934]	0.776 [0.0882, 0.937]	0.742 [0.128, 0.927]	0.787 [0.0882, 0.939]	0.775 [0.128, 0.934]

Table showing baseline(BL) and endpoint (EP) values for each neurogenesis marker in each intervention group.

Table S4 *– Blood-based biomarkers at baseline and endpoint.*

	Active		Placebo		Overall	
	**BL (N=49)**	**EP (N=49)**	**BL (N=55)**	**EP (N=55)**	**BL (N=104)**	**EP (N=104)**
**GFAP**						
Mean (SD)	108 (59.5)	112 (52.6)	128 (61.0)	128 (65.5)	119 (60.8)	120 (60.1)
Median [Min, Max]	94.8 [33.0, 387]	92.7 [39.1, 264]	123 [19.4, 346]	116 [34.3, 297]	105 [19.4, 387]	107 [34.3, 297]
**NFL**						
Mean (SD)	20.3 (9.75)	21.2 (9.88)	21.3 (8.99)	22.9 (16.0)	20.8 (9.33)	22.1 (13.4)
Median [Min, Max]	17.4 [8.34, 63.3]	19.8 [6.03, 60.0]	20.8 [1.67, 51.9]	19.8 [8.72, 116]	19.3 [1.67, 63.3]	19.8 [6.03, 116]
**pTau217**						
Mean (SD)	0.371 (0.258)	0.384 (0.248)	0.494 (0.322)	0.472 (0.324)	0.436 (0.299)	0.431 (0.293)
Median [Min, Max]	0.300 [0.130, 1.39]	0.300 [0.130, 1.08]	0.370 [0.140, 1.39]	0.370 [0.160, 1.46]	0.335 [0.130, 1.39]	0.315 [0.130, 1.46]
**pTau231**						
Mean (SD)	10.8 (10.2)	8.75 (4.69)	8.98 (4.12)	9.20 (4.18)	9.84 (7.62)	8.99 (4.41)
Median [Min, Max]	8.80 [3.02, 69.6]	8.12 [2.71, 27.9]	8.23 [2.32, 18.2]	8.13 [2.78, 20.3]	8.54 [2.32, 69.6]	8.13 [2.71, 27.9]

Table showing baseline (BL) and endpoint (EP) values of blood-based biomarkers.

Table S5 – Beta regression results for full cohort.

SOX2	Chisq	Df	p value
**Main variables**			
Intervention	0.49	1	0.48
visit	10.01	1	0.002
Intervention:visit	16.70	1	<0.0001
**Control variables**			
Staining batch	266.87	7	<0.0001
BMI	4.16	1	0.04
education	5.99	1	0.01
Plate location	14.46	1	0.0001
Test site	2.37	2	0.30
ApoE	0.03	1	0.86

Nestin	Chisq	Df	p value
**Main variables**			
Intervention	0.58	1	0.45
visit	0.75	1	0.39
Intervention:visit	1.08	1	0.30
**Control variables**			
Staining batch	159.19	7	<0.0001
BMI	5.77	1	0.02
Risk Type	4.16	1	0.04
Plate location	7.03	1	0.008

Ki67 proliferation	Chisq	Df	p value
**Main variables**			
Intervention	0.57	1	0.45
visit	4.34	1	0.04
Intervention:visit	0.40	1	0.53
**Control variables**			
Staining batch	137.08	7	<0.0001
Test site	30.04	2	<0.0001

CC3 proliferartion	Chisq	Df	p value
**Main variables**			
Intervention	0.17	1	0.68
visit	3.73	1	0.05
Intervention:visit	0.03	1	0.86
**Control variables**			
Staining batch	273.98	7	<0.0001
Plate location	6.23	1	0.01
Test site	32.35	2	<0.0001

Ki67 differentiation	**Chisq**	**Df**	**p value**
**Main variables**			
Intervention	0.0001	1	0.99
visit	6.97	1	0.008
Intervention:visit	1.41	1	0.24
**Control variables**			
Staining batch	66.51	4	<0.0001
Risk Type	3.55	1	0.06
Test site	5.95	2	0.05

DCX	**Chisq**	**Df**	**p value**
**Main variables**			
Intervention	0.0001	1	0.99
visit	7.88	1	0.005
Intervention:visit	0.08	1	0.77
**Control variables**			
Staining batch	127.80	4	<0.0001
Test site	8.40	2	0.015

CC3 differentiation	**Chisq**	**Df**	**p value**
**Main variables**			
Intervention	0.45	1	0.50
visit	0.22	1	0.64
Intervention:visit	0.96	1	0.33
**Control variables**			
age	6.67	1	0.001
Staining batch	554.42	4	<0.0001
Test site	7.97	2	0.02

Map2	**Chisq**	**Df**	**p value**
**Main variables**			
Intervention	0.22	1	0.64
visit	0.25	1	0.62
Intervention:visit	4.16	1	0.04
**Control variables**			
Staining batch	95.14	4	<0.0001
Test site	11.80	2	0.003
Plate location	3.88	1	0.049

Tables reporting full results for each Beta-regression model aimed at determining the effect of the intervention on neurogenesis markers.

ACN intervention does not impact cognition

In each model, there was a significant positive association with vist, suggesting that, on average, participants scores improved from baseline to endpoint, irrespective of the treatment group. The distribution of this change is shown in Figure S1, where a positive change indicates an improvement from baseline to endpoint. To summarise these trends, we grouped participants as having stable, improving or declining trajectories (Table S3) for each measure and show that for CMB and DPICNACC, most participants indeed improve. In contrast, for DPICN0CC, the majority remain within 5 points of their original scores.

Figure S1 *– Change in cognitive performance throughout ACID trial*

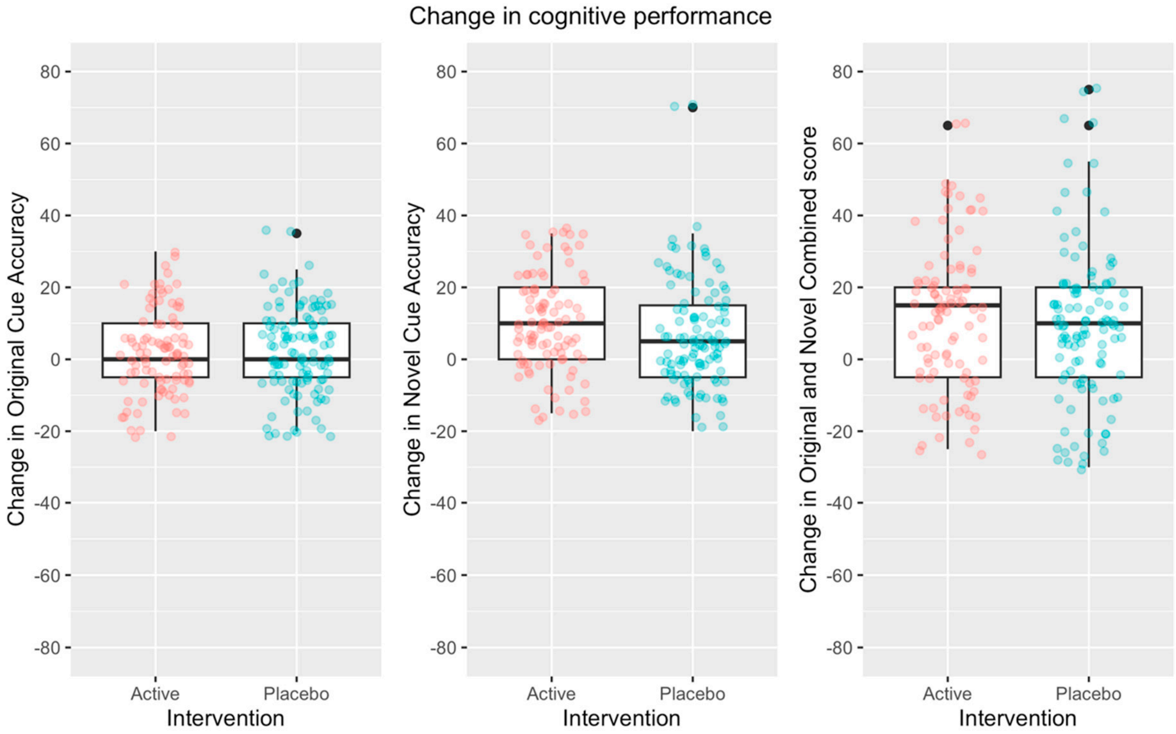

Bar graphs showing the change in each cognitive measure: original cue accuracy (DPICOACC), novel cue accuracy (DPICNACC) and original and novel combination score (CMB) across both intervention arms. A positive value indicates improvement from baseline to endpoint. A negative value indicates decline.

Table S6 *- Summary of cognitive performance trajectories.*

	**Active (N=49)**	**Placebo (N=55)**	**Overall (N=104)**
**DPICOACC**			
Declined	10 (20.4%)	11 (20.0%)	21 (20.2%)
Improved	13 (26.5%)	18 (32.7%)	31 (29.8%)
Stable	26 (53.1%)	26 (47.3%)	52 (50.0%)
**DPICNACC**			
Declined	5 (10.2%)	8 (14.5%)	13 (12.5%)
Improved	26 (53.1%)	21 (38.2%)	47 (45.2%)
Stable	18 (36.7%)	26 (47.3%)	44 (42.3%)
**CMB**			
Declined	9 (18.4%)	11 (20.0%)	20 (19.2%)
Improved	29 (59.2%)	30 (54.5%)	59 (56.7%)
Stable	11 (22.4%)	14 (25.5%)	25 (24.0%)

For the purposes of this table only, participants were classed as stable if their scores remained within a 10 point range across the two timepoints (baseline and endpoint).. Alternatively they were classed as "improved" or "declined" if their scores improved or declined by at least 5 points from baseline to endpoint.

Table S7 – Full model outputs of linear mixed effect models.

DPICOACC	Chisq	Df	p value
**Main variables**			
Intervention	0.72	1	0.40
Visit	14.36	1	0.0002
Intervention: Visit	0.19	1	0.66
**Control variables**			
age	3.10	1	0.08
education	15.05	1	0.0001
Risk Type	7.80	1	0.005
ApoE	6.08	1	0.01
DPICNACC	**Chisq**	**Df**	**p value**
**Main variables**			
Intervention	1.47	1	0.23
Visit	56.89	1	<0.0001
Intervention: Visit	0.73	1	0.3945
**Control variables**			
Test Site	7.57	2	0.02
age	18.98	1	<0.0001
CMB	**Chisq**	**Df**	**p value**
**Main variables**			
Intervention	1.99	1	0.16
Visit	45.36	1	<0.0001
Intervention: Visit	0.10	1	0.75
**Control variables**			
age	11.83	1	0.0006
education	6.41	1	0.01
Risk Type	5.48	1	0.02
ApoE	5.69	1	0.02

Results of each linear mixed effect model aimed at determining the effect of the intervention on DPIOACC, DPINACC and CMB. Full model outputs relating to Table 3.

Table S8 – Beta regression results for sub-cohort.

SOX2	Chisq	Df	p value
**Main variables**			
Intervention	0.81	1	0.37
visit	8.61	1	0.003
Intervention:visit	17.44	1	<0.0001
**BBMs**			
NFL	3.65	1	0.06
p-tau231	1.72	1	0.19
**Control variables**			
Staining batch	229.26	7	<0.0001
BMI	3.83	1	0.05
education	5.26	1	0.02
Plate location	18.65	1	<0.0001
Test site	2.75	2	0.25
ApoE	0.25	1	0.62

Nestin	Chisq	Df	p value
**Main variables**			
Intervention	1.03	1	0.31
visit	0.70	1	0.40
Intervention:visit	0.86	1	0.35
**Control variables**			
Staining batch	153.38	7	<0.0001
BMI	5.85	1	0.02
Risk Type	4.53	1	0.03
Plate location	7.03	1	0.008

Ki67 proliferation	Chisq	Df	p value
**Main variables**			
Intervention	0.23	1	0.63
visit	4.01	1	0.045
Intervention:visit	0.26	1	0.61
**Control variables**			
Staining batch	132.83	7	<0.0001
Test site	28.93	2	<0.0001

CC3 proliferartion	Chisq	Df	p value
**Main variables**			
Intervention	0.61	1	0.43
visit	3.12	1	0.08
Intervention:visit	0.01	1	0.94
**Control variables**			
Staining batch	266.52	7	<0.0001
Plate location	5.97	1	0.01
Test site	25.48	2	<0.0001

Ki67 differentiation	Chisq	Df	p value
**Main variables**			
Intervention	0.01	1	0.90
visit	8.49	1	0.004
Intervention:visit	1.05	1	0.31
**BBMs**			
p-tau231	3.80	1	0.05
**Control variables**			
Staining batch	69.85	4	<0.0001
Risk Type	2.73	2	0.26
Test site	3.71	2	0.16

DCX	Chisq	Df	p value
**Main variables**			
Intervention	0.01	1	0.93
visit	9.39	1	0.002
Intervention:visit	0.36	1	0.55
**Control variables**			
Staining batch	122.27	4	<0.0001
Test site	6.45	2	0.04

CC3 differentiation	Chisq	Df	p value
**Main variables**			
Intervention	0.50	1	0.48
visit	0.01	1	0.91
Intervention:visit	1.21	1	0.27
**Control variables**			
age	6.66	1	0.01
Staining batch	534.46	4	<0.0001
Test site	5.41	2	0.07

Map2	Chisq	Df	p value
**Main variables**			
Intervention	0.14	1	0.71
visit	0.26	1	0.61
Intervention:visit	4.42	1	0.04
**Control variables**			
Staining batch	87.20	4	<0.0001
Test site	9.76	2	0.008
Plate location	2.99	1	0.08

Tables reporting full results for each Beta-regression model aimed at determining the effect of the intervention on neurogenesis markers in sub cohort of participants with available BBM information.

Table S9 - *Mixed effect models in sub-cohort.*

DPICOACC	**Chisq**	**Df**	**p value**
**Main variables**			
Intervention	0.02	1	0.89
Visit	6.07	1	0.01
Intervention: Visit	0.36	1	0.55
**Control variables**			
education	15.44	1	0.0001
Risk Type	13.87	1	0.0002
DPICNACC	**Chisq**	**Df**	**p value**
**Main variables**			
Intervention	0.28	1	0.59
Visit	74.15	1	<0.0001
Intervention: Visit	0.66	1	0.42
**Control variables**			
age	12.39	1	0.0004
CMB	**Chisq**	**Df**	**p value**
**Main variables**			
Intervention	0.03	1	0.86
Visit	35.82	1	<0.0001
Intervention: Visit	1.69	1	0.19
**Control variables**			
age	14.73	1	0.0001

Results of each linear mixed effect model aimed at determining the effect of the intervention on DPIOACC, DPINACC and CMB in sub-cohort of participants with available BBM information

Table S10 - *Results of stratification analysis investigating the moderator effect of BMI on DPICOACC performance.*

	DPICOACC					
	**Healthy** BMI <24.9		**Overweight** BMI 25-29.9		**Obese** BMI >29.9	
	β	p	β	p	β	p
**BL cognition**	**15597**	**0.0005**	**8246**	**0.003**	**12162**	**<0.0001**
ApoE	41649	0.72	-79664	0.17	-162525	0.018
Risk Type	-175705	0.06	-93931	0.20	-75494	0.37
**CC3d_change**	**-3167074**	**0.045**	784405	0.28	**2521162**	**0.02**
Map2	989148	0.33	**-**653678	0.30	567202	0.35
Map2*ApoE	-2766561	0.09	-970339	0.30	-1058365	0.37

Left shows linear model results when analysis was restricted to individuals who were in the "healthy" BMI range (BMI of 18.5 - 24.9, n =25). Middle shows results of the analysis restricted to individuals within the "overweight" range (BMI of 25 - 29.9, n= 42) and on the right are the results of the analysis restricted to individuals in the "obese" BMI range (BMI >29.9, n=37). The estimates and p values of each variable originally included in the final model for predicting DPICOACC performance. Results show opposite CC3d associations with cognitive performance in the lowest and highest BMI groups. DPICOACC values are cubed.

Table S11 - *Results of stratification analysis investigating the moderator effect of BMI on CMB performance.*

	CMB					
	**Healthy** BMI <24.9		**Overweight** BMI 25-29.9		**Obese** BMI >29.9	
	β	p	β	p	β	p
BL cognition	**0.63**	**0.0007**	**0.62**	**0.0001**	**0.72**	**<0.0001**
ApoE	-1.02	0.91	-4.62	0.44	**-16.10**	**0.02**
Risk Type	-10.98	0.22	-3.54	0.64	-5.43	0.49
GFAP_diff	-0.14	0.23	-0.07	0.27	0.04	0.84
CC3d_change	-162.40	0.42	22.70	0.83	46.17	0.78
**CC3p_change**	**1360.68**	**0.05**	385.27	0.40	-332.91	0.47
CC3d*ApoE	-176.38	0.61	207.92	0.16	223.07	0.27

Left shows linear model results when analysis was restricted to individuals who were in the "healthy" BMI range (BMI of 18.5 - 24.9, n =25). Middle shows results of the analysis

restricted to individuals within the "overweight" range (BMI of 25 - 29.9, n= 42) and on the right are the results of the analysis restricted to individuals in the "obese" BMI range (BMI >29.9, n=37). The estimates and p values of each variable originally included in the final model for predicting CMB performance. Results show CC3p is associated with cognitive performance in only the healthy BMI group.

Table S12 - *Results of stratification analysis investigating the moderator effect of ApoE4 status on CMB performance.*

	CMB			
	**Non-ApoE4 carriers**		**ApoE4 carriers**	
	β	p	β	p
BL cognition	**0.69**	**<0.0001**	**0.60**	**0.002**
BMI	-0.51	0.31	-1.27	0.21
Risk Type	-7.79	0.10	-9.45	0.34
GFAP_diff	-0.05	0.37	-0.14	0.2
**CC3d_change**	1.09	0.99	**220.64**	**0.01**
CC3p_change	2415.66	0.18	6365.40	0.11
CC3d*BMI	-76.09	0.22	-198.13	0.16

Left shows linear model results when analysis was restricted individuals who were non-carriers for ApoE4 (n= 62). Right shows results of analysis in the subset of participants who carried one or two E4 alleles (n=42). The estimates and p values of each variable originally included in the final model for predicting DPICOACC performance. DPICOACC values are cubed. Results show Map2 is only associated with cognitive performance in ApoE4 carriers.

Table S13 - *Results of stratification analysis investigating the moderator effect of ApoE4 status on DPICOACC performance.*

	DPICOACC			
	**Non-ApoE4 carriers**		**ApoE4 carriers**	
	β	p	β	p
**BL cognition**	**12146**	**<0.0001**	**11596**	**0.001**
**BMI**	**-15735**	**0.003**	-11572	0.21
**Risk Type**	**-188833**	**0.0002**	-16171	0.86
CC3d	-6177254	0.27	-13426272	0.08
**Map2**	200715	0.55	**-1462805**	**0.01**
CC3d*BMI	246383	0.21	500041	0.05

Left shows linear model results when analysis was restricted to individuals who were non-carriers for ApoE4 (n= 62). Right shows results of the analysis restricted to individuals who carried one or two E4 alleles (n=42). The estimates and p values of each variable originally included in the final model for predicting CMB performance. Results show CC3d is only associated with cognitive performance in ApoE4 carriers.
